# Biomarker-Guided Repurposing of Chemotherapeutic Drugs for Cancer Therapy: A Novel Strategy in Drug Development

**DOI:** 10.3389/fonc.2013.00313

**Published:** 2013-12-25

**Authors:** Jan Stenvang, Iben Kümler, Sune Boris Nygård, David Hersi Smith, Dorte Nielsen, Nils Brünner, José M. A. Moreira

**Affiliations:** ^1^Faculty of Health and Medical Sciences, Department of Veterinary Disease Biology, Section for Molecular Disease Biology and Sino-Danish Breast Cancer Research Centre, University of Copenhagen, Copenhagen, Denmark; ^2^Danish Centre for Translational Breast Cancer Research, Copenhagen, Denmark; ^3^Department of Oncology, Center for Cancer Research, Herlev Hospital, University of Copenhagen, Copenhagen, Denmark; ^4^DAKO A/S, Glostrup, Denmark

**Keywords:** drug repurposing, irinotecan, TOP1, breast cancer, biomarker

## Abstract

Cancer is a leading cause of mortality worldwide and matters are only set to worsen as its incidence continues to rise. Traditional approaches to combat cancer include improved prevention, early diagnosis, optimized surgery, development of novel drugs, and honing regimens of existing anti-cancer drugs. Although discovery and development of novel and effective anti-cancer drugs is a major research area, it is well known that oncology drug development is a lengthy process, extremely costly and with high attrition rates. Furthermore, those drugs that do make it through the drug development mill are often quite expensive, laden with severe side-effects and unfortunately, to date, have only demonstrated minimal increases in overall survival. Therefore, a strong interest has emerged to identify approved non-cancer drugs that possess anti-cancer activity, thus shortcutting the development process. This research strategy is commonly known as drug repurposing or drug repositioning and provides a faster path to the clinics. We have developed and implemented a modification of the standard drug repurposing strategy that we review here; rather than investigating target-promiscuous non-cancer drugs for possible anti-cancer activity, we focus on the discovery of novel cancer indications for already approved chemotherapeutic anti-cancer drugs. Clinical implementation of this strategy is normally commenced at clinical phase II trials and includes pre-treated patients. As the response rates to any non-standard chemotherapeutic drug will be relatively low in such a patient cohort it is a pre-requisite that such testing is based on predictive biomarkers. This review describes our strategy of biomarker-guided repurposing of chemotherapeutic drugs for cancer therapy, taking the repurposing of topoisomerase I (Top1) inhibitors and Top1 as a potential predictive biomarker as case in point.

## Introduction

Despite the significant improvements in diagnosis and treatment experienced in the past few decades, cancer remains the leading cause of death worldwide, and deaths from cancer are forecasted to reach a staggering 13.2 million deaths by 2030 (1). What’s more, these numbers are only set to worsen, as a result of population aging and growth. Assuming that the estimated cancer-specific and sex-specific trends continue, it is expected that the incidence of all-cancer cases will rise from 12.7 million new cases in 2008 to 22.2 million by 2030 (2). Until now this unremitting increase has been offset by significant improvements in prognosis, as a result of earlier diagnosis, advances in surgical therapy, and the use of radiation therapy and adjuvant systemic treatments; as a consequence the survival rates for most cancers have increased significantly in the past few decades. But, unless novel and dramatically improved therapies are introduced, this compensation is unlikely to persist (2, 3). This is particularly crucial for metastatic disease as, for the large majority of cancers, it presents the biggest problem to medical management, being the main cause of death of cancer patients. In recent years our understanding of cancer biology has improved significantly, and resulted in the development of new targeted anti-cancer therapies such as targeting of the EGF-receptor or VEGF. In spite of the initial hope that agents targeting molecular alterations underlying cancer genesis and progression would provide unparalleled therapeutic benefit, reality proved otherwise. Overall, targeted therapies have shown relatively modest clinical benefit, presumably due to intrinsic resistance of tumors to inhibition of signaling intermediates, due mainly to redundancy in signaling pathways in cancer cells (4–8). As a consequence, these novel treatment modalities are not single-agent treatments as they, most often, are combined with conventional cytotoxic drugs. In short, many of the currently available molecular targeted cancer drugs are very costly, provide modest improvements in overall survival, and have significant side-effects.

## Repurposing of Cancer Drugs as a Drug Development Strategy

Although there is an acute need for developing new and better anti-cancer drugs, the lengthy time and astronomical high costs associated with cancer drug development, together with high failure rates and limited efficacy of targeted drugs have necessitated alternative approaches to cancer drug discovery (9). Drug repurposing or repositioning is a promising approach to identify suitable drug candidates for treatment of cancer; essentially it entails finding novel therapeutic indications for already approved drugs (10–14). Departing from this drug development strategy, our laboratory developed a simplified variant to identify novel therapeutic indications for chemotherapeutic agents. Our approach differs from the traditional view of drug repurposing in that we do not investigate established target-promiscuous non-cancer drugs for anti-cancer activity (10, 15, 16), but rather try to find new cancer indications for conventional chemotherapeutic agents. Most types of conventional chemotherapy are considered to kill cancer cells not by one single mechanism but by affecting several pivotal pathways/mechanisms with the sum of cellular effects resulting in cancer cell death. Accordingly, the efficacy of any given chemotherapeutic agent may be difficult to foretell using a single molecular predictor. On the other hand, some key molecules have been identified as major targets for chemotherapy drugs. With the above in mind, one could initiate systematic analyses of gene aberrations, mRNA expression, and/or protein determinations of known key target molecules for given chemotherapeutic drugs, specifically in those cancer types that are not conventionally offered these drugs – a knowledge-driven repurposing strategy.

As the response rates to a specific chemotherapeutic drug might be relatively low in an unselected pre-treated patient population, it is a pre-requisite, that the repurposing strategy includes pre-selection of those patients with a favorable molecular profile in their cancer cells, i.e., those patients with the highest likelihood of obtaining benefit from the treatment. One reasonable assumption would be that one and the same molecule would be both a major target for a chemotherapeutic drug and a predictive biomarker, a hypothesis that is supported by recent evidence. For example, two meta-analyses recently concluded that breast cancer patients with amplification of the topoisomerase 2A (*TOP2A*) gene have more clinical benefit from treatment with topoisomerase II inhibitors than patients with normal *TOP2A* gene number in their cancer cells (17–19). In the present review, we first describe and discuss experiences with topoisomerase I (Top1) measurements in colorectal cancer (CRC). We then turn to a discussion of the repurposing of DNA Top1 inhibitors for treatment of breast cancer.

## Repurposing of Top1 Inhibitors – Irinotecan

Several classes of cytotoxic agents, such as Top1 inhibitors (irinotecan, topotecan), topoisomerase II inhibitors (etoposide), anthracyclines (epirubicin, doxorubicin, mitoxantrone), taxanes (docetaxel, paclitaxel), anti-mitotics (vinorelbine, eribulin), antimetabolites (capecitabine, gemcitabine), or platinum analogs (cisplatin, carboplatin) can be used for the treatment of cancer, be it in the adjuvant, neoadjuvant, or metastatic setting. Each drug class, in addition to a specific therapeutic profile, has its own characteristic toxicity profile. The interplay of these two parameters determines the clinical use of any given drug class, which in many cases is disease specific. As a consequence, in current clinical practice, various drugs are used following evidence-based recommendations for each cancer type; for instance, drugs such as taxanes and anthracyclines are commonly used for standard treatment of breast cancer but not CRC (20). Conversely, camptothecins are used for standard treatment of CRC but not breast cancer (21). These differences in standard clinical use of chemotherapeutic agents essentially reflect the magnitude of clinical benefit attained by the different drugs in clinical trials for each specific disease. One class of anti-cancer drugs of particular interest to us is that of Top1 inhibitors, in particular irinotecan. Irinotecan is a derivative of camptothecin, and it has a unique pharmacological profile, as Top1 is its only target (22), and therefore an obvious candidate for our knowledge-driven repurposing strategy.

Irinotecan is a prodrug, 7-ethyl-10-[4-(1-piperidino)-1-piperidino] carbonyloxycamptothecin (CPT-11), which is converted by carboxylesterases into its active metabolite, 7-ethyl-10-hydroxycamptothecin (SN-38), a potent Top1 inhibitor (23, 24). SN-38 functions by inhibiting the Top1 enzyme, which plays an essential role in alleviating the topological stresses that arise during DNA replication and transcription by nicking, relaxing, and re-ligating the double-stranded DNA structure (22). The current model for anti-cancer activity by irinotecan revolves around the stabilization of (normally) transient DNA-Top1 complexes (termed “cleavage complexes” or Top1cc) by SN-38, thereby inhibiting subsequent re-ligation of the nicked DNA strand. Following the collision of DNA or RNA polymerases into the SN-38-stabilized Top1cc, DNA damage occurs. It has been suggested that upon collision with a DNA polymerase, double-strand breaks are formed, whereas RNA polymerase collision causes the formation of irreversible Top1cc-associated single strand breaks (22, 25, 26). Unless repaired, this DNA damage can lead to cell death [reviewed in (27)].

## Top1 Inhibitors in Routine Cancer Treatment

Irinotecan and topotecan are the two Top1 inhibitors routinely used in cancer treatment (Table 1). In both Europe and the United States (US) irinotecan is recommended by national guidelines as first or second line treatment for metastatic CRC (28, 29). Recently, the combination of 5 FU, irinotecan, and oxaliplatin (FOLFIRINOX) has been recommended in European guidelines on pancreatic cancer, for patients with metastatic disease, ≤75 years of age with a good performance status (30). American guidelines, however, do not recommend the use of irinotecan for the treatment of advanced pancreatic cancer (31). A new liposomal formulation of irinotecan (MM-398) has recently been tested in a large phase II study in patients with metastatic pancreatic cancer. Patient recruitment has been completed, however, no results have been published yet (32).

**Table 1:** **Approved and recommended indications for the use of irinotecan and topotecan**.

	Europe	United States
**IRINOTECAN**		
Metastatic colorectal cancer	X	X
Metastatic pancreatic cancer	X	
Metastatic small-cell lung cancer	X	X
**TOPOTECAN**		
Metastatic ovarian cancer	X	X
Metastatic cervical cancer	X^a^	X
Metastatic small-cell lung cancer	X	

*^a^Approved by authorities but not recommended in clinical guidelines*.

Topotecan is recommended for later line treatment of metastatic ovarian cancer in both Europe and the US (33, 34). Also in both Europe and the US topotecan in combination with cisplatin is approved for the treatment of recurrent cervical cancer (35, 36). Although European guidelines refer an overall survival advantage with topotecan in combination with cisplatin compared to monotherapy, combination therapy with topotecan is not recommended for the treatment of metastatic cervical cancer (37). European guidelines on small-cell lung cancer (SCLC) recommend combinations of irinotecan–cisplatin, or topotecan–cisplatin as alternative treatment options for metastatic disease in the case of contraindications to etoposide (a topoisomerase II inhibitor) (38). US guidelines recommend irinotecan combined with cisplatin (among other regimens) as first line treatment for metastatic SCLC and irinotecan monotherapy as second line therapy (39).

A search on clinicaltrials.gov revealed that irinotecan and topotecan alone or in combination with other drugs, currently are being investigated for numerous other indications including various brain tumors, sarcomas, non-small-cell lung cancer (NSCLC), triple negative breast cancer, and gastric, esophageal, and gastroesophageal junction cancers (32). Finally, etirinotecan is a new polymer conjugate of irinotecan (NKTR-102). This drug formulation has a half-life of approximately 50 days compared to 5 days for irinotecan and has shown a lower maximum concentration resulting in greater systemic exposure to SN-38 compared to irinotecan (40). It is currently investigated for the indications SCLC, NSCLC, glioblastomas, and breast cancer (32, 41, 42).

## Topoisomerase I Gene Structure, Expression, and Activity in Cancer

The topoisomerase I (*TOP1*) gene is located at 20q12, a region that frequently undergoes copy-number alterations across cancer types, including melanoma, breast, colorectal, ovarian, and gastric cancer (43–47). These copy-number alterations have been reported to occur as either gains of chromosome 20, 20q, or as amplification of smaller chromosomal regions, termed “amplicons.” Research suggests that in CRC, *TOP1* copy number increases occur predominately in conjunction with the rest of 20q (44–48). Amplification of the *TOP1* gene is observed in a subset of *TOP1* gains, and interestingly, these two types of copy number increases appear to have differential prognostic effects in stage III CRC patients (49). We have recently applied a *TOP1*/CEN-20 fluorescence *in situ* hybridization (FISH) probe mixture to explore the *TOP1* gene copy numbers in stage III CRC (44, 48). The *TOP1* and CEN-20 signals from unaffected epithelial mucosa (*n* = 50) located adjacent to the tumor cells were applied to determine the diploid copy numbers in non-cancer cells. Based on these non-cancer signals we found that 84% of the tumor samples demonstrated an increased *TOP1* gene copy number and 64% had an increased *TOP1*/CEN-20 ratio compared with the non-affected mucosa (44). Of the 50 stage III CRC patients, 13 (26%) had more than 4 *TOP1* copies/cells and 16 (32%) had a *TOP1*/CEN-20 ratio above 1.5 (44). In another study we included 154 stage III CRC chemonaïve patients and found that 55 (35.7%) of the tumors had an increased *TOP1* copy number above 4*n* gene copies per cell and 44 (28.6%) had a *TOP1*/CEN-20 ratio above 1.5 (48). There was no significant correlation between the *TOP1* copy number and proliferation, while multivariate analyses demonstrated a prognostic value since the *TOP1* copy number was significantly associated with overall survival (48). In gastric cancer, several amplicons have been observed on 20q, including one encompassing the *TOP1* gene (43). In malignant melanoma, high level amplifications of the *TOP1* locus can be detected by FISH, indicating the presence of an amplicon, which includes *TOP1* (45). In breast cancer, several amplicons mapped to 20q have been identified, including one covering the 20q12-q13 region (46). By FISH analyses we have established the normal range of *TOP1* copy numbers and found that 31% of primary breast cancer patients have *TOP1* copy number gains (≥4 copies) (50). However, it does not appear that *TOP1* is part of the minimal common region of amplification, indicating that its amplification may occur as a passenger to events involving of an amplicon located at 20q13.1–q13.2 (51). A similar finding has been made in ovarian cancer (47). Taken together, the *TOP1* locus appears to undergo frequent copy number increases in several cancer types. These aberrations are either focal in nature, i.e., amplicon-driven, or may involve larger chromosomal regions, such as 20q. Numerous candidate oncogenes located on 20q have been suggested as the targets of these copy-number alterations. Putative targets include *BCL2L1* (20q11.21), *AIB1* (20q12), and *AURKA* (20q13.2), which have all been implicated in cancer (43, 52, 53). Whether *TOP1* is truly the target of these copy number increases, or whether these increases occur as passenger-related events targeting alternative oncogenes, remains to be elucidated.

Beyond the *TOP1* copy-number alterations at the genomic level, there is also frequent over-expression of *TOP1* mRNA, Top1 protein or enzyme activity level in various cancer types compared to normal adjacent non-cancerous tissue (54–56). Generally, there appears to be a positive correlation between gene expression level, protein level, and activity in cancer tissues (54–56).

### Colorectal cancer

Colorectal cancer is the most thoroughly examined cancer with regard to Top1 expression and several studies have found increased Top1 protein in CRC tissues compared to non-cancerous tissues. Already in 1989, immunoblot analyses were applied to show that Top1 protein levels were 14- to 16-fold higher in primary colon adenocarcinoma tissue (*n* = 38) than in normal colonic mucosa (57). Approximately 20–30% of the tumors presented with very high levels of Top1 expression, whereas all normal tissue samples had low levels. Subsequent studies have largely confirmed these data finding 2- to 40-fold increases of *TOP1* mRNA, Top1 protein, or activity (55, 58) in cancer tissue. Copy-number analyses showed that *TOP1* was amplified in 23% of Dukes’ C CRC patients (*n* = 52) when compared to paired normal colon tissue and these *TOP1* amplified tumors had approximately two-times higher RNA level and protein expression level than did the diploid tumors (56). The enzyme activity of Top1 has also been evaluated in crude nuclear extracts from CRC and normal tissue. These data showed that the Top1 activity was significantly higher in primary tumor tissue compared to normal tissue (*n* = 53) (59). In concordance with Giovanella et al. (57), it was found that 20–30% of the tumors possessed very high Top1 activity although the coefficients of variations in these analyses were about 75–80% indicating that these data may be somewhat ambiguous.

Studies have also compared the Top1 protein levels and activity in metastatic CRC tissue to normal tissue and to primary CRC tissue. These data are so far inconclusive. Apparently, the Top1 activity was significantly lower in liver metastases than in the normal liver (*n* = 8) (59). The *TOP1* mRNA levels in FFPE samples did not show significant changes when comparing primary CRC tumor and liver metastasis (*n* = 33) (60) whereas the levels of Top1 protein expression were higher in malignant cells from tumor recurrences compared to primary tumors (*n* = 40) (61) and *n* = 25 (62). Yet another study found concordance between Top1 protein levels in paired primary CRC and lymph node metastases in 33 of 42 cases (63).

Other studies have investigated the protein levels of Top1 in primary tumor CRC tissue by Immunohistochemistry (IHC). These studies have found high Top1 expression in 45% (*n* = 62) of metastatic CRC patients that received a first line 5 FU/CPT-11 chemotherapy (64), 86% (*n* = 29) of primary colon cancers (65), 31% (*n* = 13) among patients with recurrent CRC (66), 17% (*n* = 1,313) in metastatic CRC patients (67), and a study comprising 498 Dukes’ stage B and C patients reported positive/high Top1 protein expression in 48% of the cases (68). These differences may be due to differences among the studied patient cohorts, choice of antibodies, tissue micro arrays (TMAs) versus full section analyses and scoring systems. In brief, *TOP1* mRNA, Top1 protein, and activity are increased in CRC tissues in comparison to non-cancerous tissues and a substantial subgroup of CRC patients has high levels of Top1.

### Other cancers

Elevated levels of Top1 have also been reported in various other cancers. In poorly differentiated ovarian carcinomas the activity of Top1 was found to be much higher than in non-cancerous tissue or benign tumors (54, 69). In support of this, IHC analyses demonstrated that Top1 protein is primarily associated with tumor cells and much less to normal infiltrating cells (70) and increased Top1 protein expression was found in 43% of ovarian carcinomas (71). Prostate tumors also possessed increased levels of Top1 protein levels and Top1 activity compared to matched non-cancerous tissues, whereas no difference between malignant and normal tissue was found in kidney tumors (55). Similar over-expression of Top1 protein have been reported in urinary bladder carcinomas (77%) (72), gastric carcinomas (68%) (73), testicular tumors (74), renal cell carcinomas (36–100%) (75), malignant melanomas (42%) (76), squamous cell carcinomas (92%) (77), and sarcomas (13%) (78). In metastatic breast cancer (mBC) the Top1 protein expression has been evaluated by IHC in FFPE tissue from 22 primary breast cancer. It was found that 41% over-expressed Top1 (79). Interestingly, the expression of Top1 protein varies from undetectable to strongly positive among the analyzed samples, which indicate that Top1 expression may be a suitable biomarker in a subgroup of mBC patients.

## Clinical Studies Evaluating the Predictive Role of Top1

Until now the association between Top1 assessed in tumor tissue and irinotecan efficacy has only been investigated retrospectively and with focus on CRC. Top1 levels have been determined by IHC where protein expression was assessed (64, 67, 68) and by RT-PCR where gene expression (mRNA) was analyzed (60). Tumor samples were obtained from patients who were originally enrolled in randomized phase III trials or from patients routinely treated in accordance with current local clinical guidelines.

Two small single-cohort biomarker studies investigated patients with advanced CRC who were all treated with different regimens of 5 FU/leucovorin + irinotecan (60, 64, 67, 68). These studies did not identify any significant association between *TOP1* gene expression or Top1 protein expression and objective response rates or survival endpoints. However, both studies were methodologically flawed as Top1 data was only available from 62 to 33 patients, respectively, and due to the consequent inherent lack of sufficient statistical power, this makes it almost impossible to obtain statistically significant results even though the association in question was in fact true. Additionally, a true distinction between a predictive and a prognostic component of a biomarker will not be identified when survival analysis is performed in a single-cohort study without a relevant control group (80, 81).

Biomarker studies designed to obtain Level of Evidence (LoE) 1 as proposed by Simon et al. (81) have been conducted where material from randomized clinical phase III trials was used in order to conduct a so-called prospective-retrospective biomarker evaluation according to a stringent analysis plan. In a study by Braun et al. (67) primary tumor material from patients originally accrued in the UK MRC FOCUS study (82) was used. The UK MRC FOCUS study was a randomized clinical trial investigating different combinations of chemotherapy for patients with advanced CRC. In first line 1,628 patients were randomized between 5 FU/levofolinate, 5 FU/levofolinate + irinotecan or 5 FU/levofolinate + oxaliplatin. As patients in the 5 FU/levofolinate arm could be used as relevant controls to correct for potential concurrent prognostic qualities of the biomarkers in question, this clinical design was ideal when investigating putative predictive biomarkers of either irinotecan or oxaliplatin efficacy. In the biomarker study, Top1 protein expression was assessed by IHC using a murine monoclonal antibody (clone 1D6, Novocastra), and the staining intensity was graded as low, moderate, or high. Due to inadequate tumor material or failed IHC analysis 315 cases were excluded, which resulted in available Top1 data from 1,313 tumor samples (81%). The authors reported a significant association between staining intensity and progression free survival where patients with tumors showing moderate or high expression benefited from the addition of irinotecan compared to 5 FU/levofolinate therapy alone. In contrast, patients with Top1 low classified tumors did not benefit more from the irinotecan combination than from the 5 FU/levofolinate treatment alone. The interaction between Top1 and the irinotecan combination was reported to be statistically significant (*P* = 0.001). An attempt to validate these results was performed in tumor material from the CAIRO trial (83). In the CAIRO trial, 820 patients with advanced CRC were originally randomized between capecitabine, a prodrug of 5 FU, or capecitabine + irinotecan as first line treatment. In the following biomarker study (84), which was only published in abstract form, tumor samples from 545 patients were included and the same methodologies as in the study by Braun et al. (67) were applied. The study failed to confirm the positive association between Top1 protein expression and irinotecan efficacy. There are several explanations to why confirmation failed. First, the hypothesis may not be correct, and second, potential methodological bias may have been introduced unintentionally. Assessment of IHC staining intensity can be problematic and inter-observer variability due to staining heterogeneity and the somewhat subjective nature of the evaluation is a challenge to this methodology. Additionally, information on analytical validation of the applied antibody is essential to ensure prober sensitivity and specificity, and to our knowledge this is lacking for the 1D6 clone, which was used in both the CAIRO and the UK MRC FOCUS trials – as a result we cannot objectively determine which trial, if any, may be at fault. However, as stated previously, both the CAIRO and the UK MRC FOCUS trials fulfill the requirements set by Simon et al. (81) in order to obtain LoE 1 for a predictive biomarker of irinotecan efficacy, and the trials still represent the best available option to retrospectively assess the association between other biomarkers or Top1 analyzed by techniques other than IHC and irinotecan in the advanced setting of CRC.

The association between Top1 protein expression and irinotecan efficacy has also been investigated in the adjuvant setting of CRC, and results from a retrospective biomarker study suggested a positive predictive role of Top1 protein expression (68). The study did not use material from one randomized clinical trial but included material from several clinical trials, which resulted in two cohorts of patients who were either treated with 5 FU/leucovorin alone or 5 FU/leucovorin + irinotecan. However, this methodology was intrinsically flawed as the original clinical trials spanned almost two decades, a time frame in where great surgical improvements in the managements of CRC have taken place.

Based on negative results from several phase III trials, i.e., the PETACC-3 (85) and the CALGB 89803 (86), the 5 FU/leucovorin + irinotecan combination is today not recommended in the adjuvant setting of colon cancer. However, as patients in these trials were randomized between 5 FU/leucovorin and 5 FU/leucovorin + irinotecan, tumor tissue from these trials is highly appropriate for retrospective biomarker research in relation to prediction of irinotecan efficacy. The main challenge with such an approach is the availability of a sufficient number of tumor samples to obtain the necessary statistical power.

## Design of Clinical Studies to Validate Predictive Biomarkers

Repurposing often involves drugs where the mechanisms of action are fully or partly known. Thus, clinical repurposing trials may take the advantage of such knowledge and from early phase development/testing include predictive biomarkers. Such biomarkers will often be found among molecules known to be mechanistically involved in sensitivity/resistance to the drug. The use of predictive biomarkers in early drug testing may increase the therapeutic index of the drug in question by increasing the efficacy of the drug in the selected biomarker favorable population and at the same time avoid drug-induced toxicity in the biomarker unfavorable population as these patients will not be exposed to the drug. Looking ahead, future drug indications might be limited to small subgroups of patients based on predictive biomarkers. Targeted drug selection is already in routine use e.g., estrogen receptor and human epidermal growth factor receptor (HER) 2 in breast cancer, *KRAS* in CRC and *BRAF* in malignant melanomas. The status of the relevant marker is frequently based only on analysis of the primary tumor. However, in e.g., breast cancer accumulating evidence suggests that tumor characteristics, including ER and HER2 might change through tumor progression (87). Thus, the treatment strategy may require readiness to perform serial biopsies, including biopsies from metastatic lesions. The statistical considerations and principles for repurposing an old drug accompanied by a biomarker are exactly the same as for the development of a new (targeted) drug.

### Phase I

Most often, repurposing of an old drug will not involve a phase I trial. However, new knowledge concerning relevant biomarkers might encourage the clinicians to try new drug combinations and thus perform a phase I trial.

The goal of incorporating biomarkers in this stage of development is a better characterization of the biomarker and the assay performance in human samples (88). In this context the present EMA guidelines urge investigators of non-cytotoxic products to analyze not only biopsies from the primary tumor and metastasis but also normal tissue to understand the molecular background for efficacy (89). More recently, molecular pre-screening has been suggested for selecting patients for early drug development. Thus, it is envisioned that academic institutions establish molecular pre-screening programs in order to select patients for phase I trials (90).

### Phase II

The biomarker should be included for hypothesis testing and early indications for proof-of concept. There are two types of clinical trial designs effective in evaluating the role of a potential predictive biomarker in phase II: the adaptive parallel two stage design and the tandem two-step predictor biomarker evaluation trial design. The designs and rational behind them have been reviewed by McShane et al. (88).

### Phase III

Phase III studies designed to repurpose an old drug will most often involve late stage cancer patients in order to compare monotherapy with a test drug versus best supportive care. Alternatively, the test drug might be evaluated as an add-on to a known treatment.

Prospectively designed clinical trials are regarded as the gold standard for evaluating a predictive biomarker. In many instances, however, due to time and expenses required for these trials, a retrospective testing of predictive biomarkers is more feasible.

Retrospective validation of biomarkers is regarded as an acceptable strategy in selected circumstances. The strategy requires data from well-designed prospective phase III, randomized trials, sample availability from on a large majority of patients to avoid bias due to patient selection, a prospectively stated hypothesis, a predefined and standardized assay, and upfront sample size and power justification (91). Optimally, evidence should be provided from two independent randomized trials. *KRAS* as a predictor for efficacy of cetuximab and panitumumab in CRC is an example of a biomarker which has successfully been validated using a retrospective strategy.

In general there are four types of clinical trial designs to evaluate a potentially predictive biomarker: (1) the all-comers design with a “biomarker end point” as second objective, (2) a targeted design that restricts the study population to patients who have a favorable predictive biomarker profile, (3) a strategy design which randomizes patients to receive biomarker-based or non-biomarker-based (standard) treatment, and (4) a multiple hypothesis design, which combines the targeted design and the all-comers design. The latter design addresses the multiple hypotheses by having co-primary objectives (91, 92). Each of the designs has potential advantages and disadvantages. The all-comers design requires validation in a separate trial while the other designs prospectively evaluate the biomarker. Choice of design should depend upon knowledge on the biomarker and disease setting (91–94).

The REporting recommendations for tumor MARKer prognostic studies (REMARK) guidelines were developed in order to standardize and improve the quality of cancer biomarker studies. Reporting of results should follow these guidelines (95). More recently, guidelines for conducting experiments using tissue microarrays have been published (96). This checklist should be used in addition to the REMARK guidelines. With a more rational drug development including biomarker driven trials, researchers might ultimately yield greater benefits for patients.

## Repurposing Irinotecan to Breast Cancer

Breast cancer is the most common kind of cancer among women. Improved adjuvant treatment in early breast cancer has resulted in better prognosis, but still approximately 20% of women, initially diagnosed with regional disease will develop systemic recurrence within 5 years.

Two major, still unresolved, medical problems are that almost all patients with mBC who obtain an objective response to chemotherapy will eventually experience disease recurrence and death from their disease. Secondly, a large fraction of the patients with mBC who receive first line systemic chemotherapy will not gain any beneficial effects from the treatment. In contrast, they may suffer from drug-induced side-effects and in addition, initiation of a potential effective second line treatment may be delayed until lack of response to the first line treatment is evidenced.

In current treatment of mBC, the main first line cytotoxic drugs are anthracyclines, and/or taxanes combined with cyclophosphamide. Second line treatment may include 5 FU, gemcitabine, platin derivatives, or vinorelbine. Unfortunately, very few options are available as third line treatment. It is thus clear that there is an urgent need for new and effective drugs in this setting. On the other hand, such drugs should be used with caution as they may be associated with significant side-effects with severe influence on the quality of life of the patients. If possible, such drugs should be used in combination with predictive biomarkers, allowing for a personalized treatment approach in which only patients with a high likelihood of an objective response should be offered the treatment in question. A number of publications have demonstrated some benefit from irinotecan treatment in patients with mBC being refractory to current breast cancer treatment (21). However, with a relatively small group of patients obtaining benefit from the treatment and the rather serious side-effects associated with irinotecan treatment, there will be a need for a predictive biomarker profile when introducing irinotecan in the treatment of mBC. We describe here, using the example of repurposing of Top1 inhibitors for the treatment of breast cancer, our approach to identify novel therapeutic indications for standard chemotherapeutic agents, based on prior knowledge of the pharmacology of these agents and exploratory studies for biomarker establishment.

**Figure 1:**
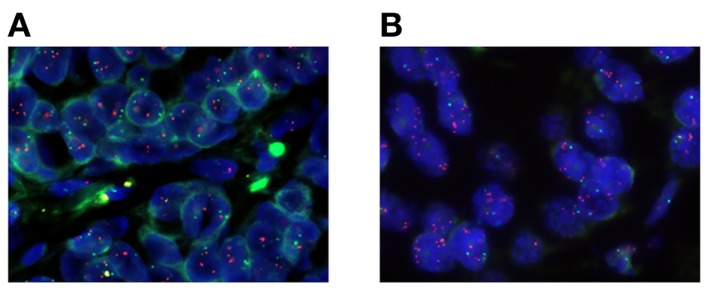
**Microscope photographs of two different primary breast cancer specimens stained with a fluorescent *TOP1*/CEN-20 fluorescence *in situ* hybridization (FISH) probe mix**. Red spots visualize the *TOP1* gene and green spots represent CEN-20. **(A)** A breast cancer specimen with diploid *TOP1* copy number of 2.97, CEN-20 copy number of 1.90, and a ratio of 1.56. **(B)** A breast cancer specimen with amplified *TOP1* copy number of 6.35, CEN-20 copy number of 1.90, and a ratio of 3.34.

The gene expression level of *TOP1* may not always predict response to camptothecin (97, 98) and the currently available antibodies to the Top1 protein have not yet been sufficiently validated. FISH is a validated clinical method to be used on FFPE tissue and it provides a direct measure of cancer cell gene aberrations on a cell to cell basis and may therefore provide more specific information than global genomics techniques. Therefore, we have used a *TOP1*/CEN-20 FISH probe mix to determine the *TOP1* gene aberration frequency in clinical breast cancer biopsies (*n* = 100) and compared to findings in normal breast tissue (*n* = 100). These data demonstrated that *TOP1* gene copy numbers of normal breast tissues were all in the diploid range, whereas 31% of the breast cancer samples had *TOP1* copy number gain (≥4 copies) (50). In breast cancer tissue we have observed a significant association between the *TOP1* copy numbers and the *TOP1* mRNA expression (50) which in combination with the frequent amplification of the *TOP1* gene suggest that *TOP1* gene copy numbers may be clinically relevant as a potential predictive biomarker for irinotecan sensitivity in breast cancer. Based on our FISH data and published reports on the response rates of irinotecan in mBC (21), we have initiated two clinical phase II trials with mBC patients being refractory to anthracyclines and taxanes. Patients with *TOP1* copy number gain (≥4 copies) are offered treatment with irinotecan. The patients are stratified according to HER2 levels being either HER2-positive (POSIRI; EudraCT 2012-002347-23) or HER2-negative (NEGIRI; EudraCT and 2012-002348-26). The main goal is to get objective response rate according to RECIST 1.1. In Figure 1 we have exemplified our approach to biomarker-guided repurposing of irinotecan in breast cancer by picturing two individual ER-positive and HER2-neutral breast cancer patients. These patients possess either *TOP1* copy numbers in the normal range (Figure 1A) or increased *TOP1* copy numbers (Figure 1B) and only the latter would therefore be eligible for irinotecan therapy. If these studies and a subsequent phase III trial are positive, the *TOP1* copy number may be applied as a predictive biomarker for irinotecan treatment in anthracycline and/or taxane refractory mBC. Additionally, an association between *TOP1* copy numbers and irinotecan effect should subsequently be tested in the other cancer types not currently being treated with irinotecan. We believe that the workflow described here can be applied to other chemotherapeutic drugs and/or other indications, providing a viable shortcut to novel effective treatments.

## Conclusion

Currently, few people would argue against that the future of drug development in oncology lies with the identification of predictive biomarkers capable of identifying those subsets of patients who will benefit from a given therapy. The use of biomarkers to pinpoint those with a favorable response profile, normally a small subgroup of patients, within a large population is at the heart of the concept of personalized medicine. Also, the use of companion molecular diagnostics promise to minimize the size, costs, and failure rates of cancer agents in clinical trials.

We describe here our strategy of biomarker-guided repurposing of chemotherapeutic drugs for cancer therapy, exemplified with the repurposing of Top1 inhibitors and Top1 as a potential predictive biomarker. This approach can conceivably be implemented to a substantial number of currently used chemotherapeutic drugs, since their mechanisms of action are well studied with thousands of studies available in the literature. We believe that this strategy is valuable and can, potentially, add new tools to the armamentarium of drugs at the disposal of oncologists.

## Conflict of Interest Statement

The authors declare that the research was conducted in the absence of any commercial or financial relationships that could be construed as a potential conflict of interest.
